# Fluids and body composition during anesthesia in children and adolescents: A pilot study

**DOI:** 10.1007/s00431-024-05490-x

**Published:** 2024-02-26

**Authors:** Céline Betti, Ilaria Busi, Cinzia Cortesi, Luciano Anselmi, Mario Mendoza-Sagaon, Giacomo D. Simonetti

**Affiliations:** 1 Pediatric Institute of Southern Switzerland, Ospedale San Giovanni, Ente Ospedaliero Cantonale, Bellinzona, Switzerland; 2 Faculty of Biomedical Sciences, Università della Svizzera Italiana, Lugano, Switzerland

**Keywords:** Children, Impedance, Total body water, Anesthesia

## Abstract

The purpose of this study is to evaluate the intracellular and extracellular volume before and after anesthesia in order to ascertain their variations and determine the potential utility of this information in optimizing intraoperative fluid administration practices. A bioimpedance spectroscopy device (body composition monitor, BCM) was used to measure total body fluid volume, extracellular volume, and intracellular volume. BCM measurements were performed before and after general anesthesia in unselected healthy children and adolescents visiting the Pediatric Institute of Southern Switzerland for low-risk surgical procedures hydrated with an isotonic solution. In 100 children and adolescents aged 7.0 (4.8–11) years (median and interquartile range), the average total body water increased perioperatively with a delta value of 182 (0–383) mL/m^2^ from pre- to postoperatively, as well as the extracellular water content, which had a similar increase with a delta value of 169 (19–307) mL/m^2^. The changes in total body water and extracellular water content significantly correlated with the amount of fluids administered. The intracellular water content did not significantly change.

*  Conclusion*: Intraoperative administration of isotonic solutions results in a significant fluid accumulation in low-risk schoolchildren during general anesthesia. The results suggest that children without major health problems undergoing short procedures do not need any perioperative intravenous fluid therapy, because they are allowed to take clear fluids up to 1 h prior anesthesia. In future studies, the use of BCM measurements has the potential to be valuable in guiding intraoperative fluid therapy.

**Table:** 

**What is Known:** *• Most children who undergo common surgical interventions or investigations requiring anesthesia are nowadays hydrated at a rate of 1700 mL/m*^*2*^*/day with an isotonic solution.* *• The use bioimpedance spectroscopy for the assessment of fluid status in healthy children has already been successfully validated.* *• The bioimpedance spectroscopy is already currently widely used in various nephrological settings to calculate fluid overload and determine patient’s optimal fluid status.*	
**What is New:** *• Routine intraoperative fluid administration results in a significant fluid accumulation during general anesthesia in low-risk surgical procedures.* *• This observation might be relevant for children and adolescents with conditions predisposing to fluid retention.* *• In future studies, the use of BCM measurements has the potential to be valuable in guiding intraoperative fluid therapy.*

**Supplementary Information:**

The online version contains supplementary material available at 10.1007/s00431-024-05490-x.

## Introduction

Hypotonic intravenous fluid maintenance therapy with 5% dextrose at a rate of 1700 mL/m^2^/day (i.e., 100 mL/kg/day for the first 10 kg of weight, 50 mL/kg/day for the next 10 kg of weight, and 20 mL/kg/day for weight over 20 kg) supplemented with sodium 3 mmol/kg/day has been routinely prescribed to children who undergo common surgical interventions or investigations requiring anesthesia. Some years ago, it was speculated that this traditional hydration practice, first proposed by Holliday and Segar in 1957, may result in hyponatremia and in an increased intracellular water content [1]. The suggestion was followed by a fascinating and often heated controversy. The debate has quieted in the meantime: well-designed trials comparing the use of hypotonic versus normal saline (0.9%) or lactated Ringer demonstrate that hypotonic fluids cause acute hyponatremia, whereas normal saline or lactated Ringer effectively prevents it. As a consequence, most children who undergo common surgical interventions or investigations requiring anesthesia are nowadays hydrated at the abovementioned rate with an isotonic solution such as normal saline or lactated Ringer [2].

The amount of sodium administered using normal saline or lactated Ringer is about 3 times higher than that administered with the traditional Holliday-Segar method [3]. It is therefore tempting to assume that this strategy may sometimes be associated with an extracellular fluid volume expansion.

Bioimpedance devices use a whole-body bioimpedance spectroscopy technique, which is commonly used for the evaluation of body composition, total body fluid volume, intracellular volume, and extracellular volume, both in adults and children [4–6]. The bioimpedance spectroscopy is already currently widely used in various nephrological settings to calculate fluid overload and determine patient’s optimal fluid status [7–10] and its use for the assessment of fluid status in healthy children has already been successfully validated [11].

The aim of the present study was to determine pre- to postoperative fluid distribution with a bioimpedance device in both children and adolescents undergoing low-risk surgical interventions requiring general anesthesia. The goal of this investigation is to better understand the water content, the body composition, and the fluid status before and after anesthesia in order to define a better intraoperative fluid administration, which has never been assessed in previous studies in children and adolescents with the bioimpedance technique.

## Methods

We performed a prospective, observational, single center, cohort study in a pediatric population aged 0–16 years visiting the Pediatric Institute of Southern Switzerland, EOC, site of Ospedale San Giovanni in Bellinzona.

### Patients

The body composition monitor (BCM, Fresenius Medical Care, Germany) was used for measurements, which were consecutively performed immediately before and after anesthesia in 100 unselected children and adolescents for both inpatient and outpatient surgery procedure. All patients had an ASA (American Society of Anesthesiology) physical status classification system score of ≤ ASA II, indicating an absence of severe systemic disease. Patients with a limb amputation or a congenital malformation were not measured, as well as patients who had significant blood loss and therefore had received erythrocyte concentrates or colloid solution. All patients fasted overnight for more than 6 h, but clear liquids (e.g., water, tee, syrup) were offered and suggested up to 1 h before anesthesia (and first measurement). The type and duration of anesthesia as well as the type and amount of intravenous fluid therapy were prescribed according to standard clinical practice and the most recent guidelines [2].

The study was approved by the local ethics committee and fulfilled the last version of the Declaration of Helsinki [12]. The parents of all patients gave written informed consent.

### BCM measurements

The necessary equipment includes the BCM device and four non-recyclable electrode strips. Adequate preparation involves confirming the proper calibration of the BCM device and maintaining a controlled temperature environment, a priori particularly relevant in the operating room setting. Participant instructions emphasize standardized clothing and removal of metal objects, consistent with routine pre-operative procedures. Participants are positioned in a relaxed, supine state and the immobility is facilitated by general anesthesia.

Therefore, according to the manufacturer’s guidance, four non-recyclable electrode strips were taped to wrist, hand, ankle, and foot, on the same side of the patient, lying in a supine position. Electrodes were connected to the BCM device using the cable provided by the manufacturer. Body weight, height, patient age, patient gender, and the actual blood pressure measurement were entered into the BCM device. The bioimpedance measurement itself requires approximately 2 min, is non-invasive, safe, and painless, and is possible from the neonatal age. It measures at 50 frequencies over a range from 5 to 1000 kHz to determine the electrical resistances of the total body water and the extracellular water between electrodes. Participants are positioned in a relaxed, supine state and the immobility achieved with the procedural sedation. This allows for a simple, non-invasive, objective, and accurate assessment of an individual patient’s fluid status (overhydration, total body water, extracellular, and intracellular water) and an assessment of the body composition (lean tissue mass and adipose tissue mass, expressed as lean and fat tissue mass index, i.e., tissue masses normalized to height squared (kg/m^2^)) [5].

### Other measurements

Age, height, body weight, pre- and postoperative heart rate, blood pressure, respiratory rate, oxygen saturation measurements, fluid balance data, and surgical data were documented in all patients. Weight and height were measured with the use of electronic scales (at 0.1 kg) and fixed stadiometers (at 0.1 cm). The body mass index (BMI) was calculated as weight divided by the height squared (kg/m^2^). BMI percentiles were expressed based on the CDC (Centre of Disease Control) reference intervals (overweight 85–95th percentiles; obese, > 95th percentile).

### Sample size

Data were collected in a coded manner through a specific data collection form and reported in an electronic database. To have an 80% chance of detecting as significant (at *p* < 0.05) a variation in water content of 3% from pre- to post-anesthesia values, at least 90 patients were required.

### Statistical analysis

All continuous data are presented as median and interquartile range. Changes in BCM-derived measurements from pre- to postoperatively (delta values) were obtained by subtracting the preoperative value from the postoperative value and expressed in mL/m^2^. These changes were subsequently evaluated using the Wilcoxon matched-pairs signed rank test. Linear simple regression analyses were conducted to explore the relationships between variables (dependent variables, delta changes of the bioimpedance measurements; independent variable, perioperative fluid infusion). Comparison between different groups (i.e., obese and normal weight children) was performed using the Mann-Whitney test. Statistical significance was set at *p* < 0.05. Statistical analyses were performed with GraphPad Prism version 8.0.0, San Diego, CA.

### Missing data

Listwise deletion was used to manage missing data.

## Results

The study population comprised 100 infants and children, recruited from 03 February to 07 April 2022. In this study, comprehensive data collection and meticulous record-keeping ensured the absence of missing data, facilitating a thorough analysis of the complete dataset.

Sixty-five were females and 35 males, aged 7 (4.2–11.0) years, with a body weight of 26.8 (18.1–42.7) kg and a height of 1.27 (1.10–1.54) m (Table 1). No patients had evidence of severe systemic disease, the ASA (American Society of Anesthesiology) physical status classification system being in all patients ≤ ASA II [13, 14]. Patients were classified as not exhibiting any signs or symptoms of dehydration based on clinical assessment prior to the surgery (internal practice pediatric guidelines considering clinical parameters and vital signs). Almost all patients were premedicated prior anesthesia with oral midazolam (0.5 mg/kg, with a higher maximum dose of 10 mg, *N* = 93). At the discretion of the attending anesthesiologist, and depending on the past medical history of the patient, its characteristics, and the surgical procedure, a total intravenous anesthesia (propofol) was given intraoperatively in 94% of cases, while in 6% of cases, general anesthesia was provided by an inhalation anesthetic (desflurane or sevoflurane). The general anesthesia had a median duration of 63 (44–85) minutes. Ninety-six patients received anesthesia for a surgical intervention (pediatric surgery, *N* = 79; otolaryngology *N* = 17; orthopedic surgery *N* = 4) and 4 patients for an investigation (magnetic resonance imaging).

**Table 1:** Demographic and baseline characteristics of 100 children and adolescents undergoing common surgical interventions requiring anesthesia

***N***	100
**Males:females, *N***	35:65
**Age, years**	7.0 (4.2–11.0)
**Body weight, kg**	26.8 (18.1–42.7)
**Height, m**	1.27 (1.10–1.54)
**Body surface area, m**^**2**^	0.95 (0.75–1.32)
**Body mass index, kg/m**^**2**^	16.9 (15.2–19.9)
Overweight, *N*	14
Obese, *N*	12
**Heart rate, bpm**	90 (75–102)
**Systolic blood pressure, mmHg**	103 (93–108)
**Diastolic blood pressure, mmHg**	48 (41–61)
**Duration of surgical procedure, min**	49 (30–66)
< 60 min, *N*	64
60–120 min, *N*	26
≥ 120 min, *N*	10
**Duration of anesthesia, min**	63 (44–85)
**Type of anesthesia**	
Intravenous anesthetics, *N*	94
Inhalation anesthetics, *N*	6
**Perioperative IV fluids administered, mL/m**^**2**^	256 (178–382)
Ringer-infusion, *N*	42
Ringer-infusion and NaCl 0.9%, *N*	28
NaCl 0.9%, *N*	26
None, *N*	4

Continuous variables are reported as median and interquartile range, categorical variables are presented as counts

A prophylaxis to prevent postoperative nausea and vomiting was given in 94 cases, usually with ondansetron and dexamethasone. The median duration of the surgical procedure was 49 (30–66) minutes. In the 79 pediatric surgery patients, blood loss was irrelevant; and in the other 21 (otolaryngology and orthopedic surgery), the estimated blood loss was about 50 ml.

There were no perioperatively respiratory and cardiac complications. The median perioperative fluid therapy amounted to 225 (200–400) mL, equivalent to 256 (178–382) mL/m^2^ when adjusted to the body surface area, with four patients receiving no fluid therapy at all. Approximately half of the patients was hydrated with lactated Ringer (42%), 26% received normal saline (NaCl 0.9%), and 28% received a combination of both normal saline and lactated Ringer in their fluid therapy. The urinary output was always checked in young children at the end of all surgical procedures in the diapers and no relevant amount of urine output was observed during the procedure.

All the main vital signs routinely monitored were lower immediately after anesthesia than before, as expected by the anesthetic medications used, but always in normal reference ranges (Supplementary Table S1).

As shown in Table 2, the average total body water (TBW) increased perioperatively with a delta value of 182 (0–383) mL/m^2^ from pre- to postoperatively. The extracellular water content (ECW) had an equivalent increase with a delta value of 169 (19–307) mL/m^2^. Nevertheless, the intracellular water content (ICW) did not significantly change (delta value of 0 [− 125–158] mL/m^2^). No differences were observed between the group of patients with no relevant blood loss (*N* = 79) and the group of patients (*N* = 21) with somewhat of blood loss.

**Table 2:** Volume status of 100 children and adolescents before and after anesthesia

			**Before** (**L**)	**Delta** (**mL/m**^**2**^)	***P*-value**
**Total body water**			16.4 (11.9–25.3)	182 (0–383)	***P* < 0.0001**
**Extracellular water**			6.9 (5.1–10.6)	169 (19–307)	***P* < 0.0001**
**Intracellular water**			9.5 (6.7–14.7)	0 (− 125–158)	*P* = 0.77182

Continuous variables are reported as median and interquartile range

Delta values were calculated by subtracting preoperative value from the postoperative value

Significance between pre- and postoperative was determined by Wilcoxon matched paired *t*-test (*P* < 0.05)

**Fig. 1:**
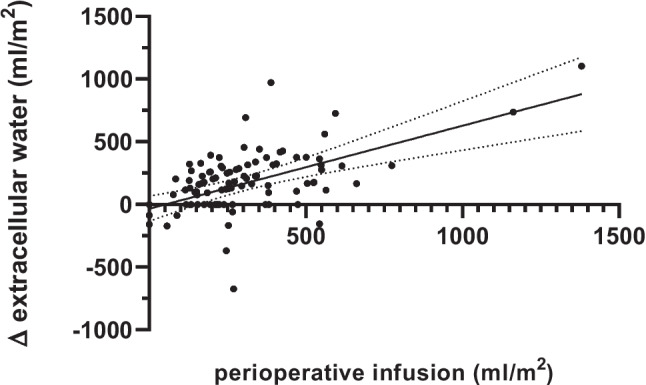
Linear correlation between fluids administered and perioperative changes in extracellular water content. The dotted lines represents the 95th confidence interval (*r*^2^ = 0.20, *p* < 0.0001; *y* =  − 35 + 0.7*x*)

Linear regression analyses evaluating the relationship between perioperative fluid administration and changes in pre- to postoperative fluid status show that the changes in TBW (*r*^2^ = 0.05, *p* = 0.02) and ECW (*r*^2^ = 0.20, *p* < 0.0001) correlate with the amount of fluids administered (Fig. 1). However, variations in ICW were not significantly associated with the fluids given.

**Fig. 2:**
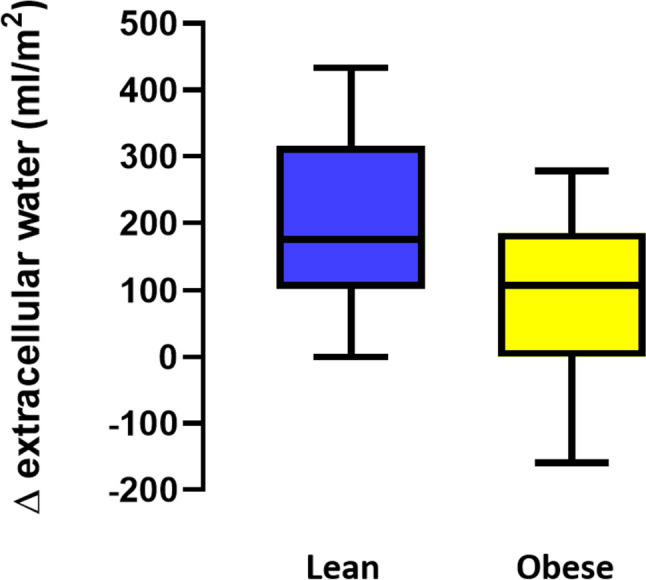
Perioperative changes in extracellular water content in lean (*N* = 74) and obese (*N* = 12) children. Box plot represents median with interquartile changes with the 10th and 90th percentile (*p* = 0.03)

Body composition, as assessed by the BCM device (fat tissue index and lean tissue index), did not show any correlation with TBW, ECW, or ICW variations. Nevertheless, comparing the group of lean children (*N* = 74) with the group of obese children (*N* = 12), lean children showed a higher perioperative increase in ECW compared to obese children (*p* = 0.03; Fig. 2) by similar administered fluids (as expressed in mL/m^2^).

## Discussion

Most children who undergo anesthesia are hydrated with an isotonic solution at a rate of 1700 mL/m^2^/day^2^. This strategy might be associated with an extracellular fluid volume expansion.

The primary findings from this preliminary investigation support the proposed hypothesis: the standard intraoperative fluid administration leads to a noteworthy elevation in both total body water and extracellular water content. The intracellular water content did not show relevant changes.

During anesthesia and perioperative circumstances, the main goals are to avoid fluid deficits, ensure electrolyte homeostasis, and achieve stability of vital parameters.

The use of normal saline (or lactated Ringer) infusions does not increase the intracellular volume (and does not cause hyponatremia anymore [3, 15]), yet provide an extracellular fluid volume expansion. This tendency is more pronounced in lean compared to obese children.

This analysis shows that the extracellular fluid expansion is directly correlated to the amount of fluid administered.

A general vasodilatation and increased secretion of proinflammatory mediators and stress hormones during surgery can be mentioned as possible explanations for this increase in total body water content. General anesthesia causes peripheral vasodilatation resulting in a redistribution and accumulation of fluids [16]. Moreover, surgery represents a traumatic insult and initiates an acute stress response leading to secretion of proinflammatory mediators and stress hormones, which control the sympathoadrenal system and the hypothalamic-pituitary-adrenal axis. Increased levels of adrenocorticotrophic hormone, antidiuretic hormone, cortisol, aldosterone, and catecholamines result in increased catabolism, reduced urinary secretion, disturbed microcirculation, and increased vascular permeability leading to salt and fluid retention [17]. An explanation for the more pronounced fluid accumulation in lean compared to obese children could be the fact that lean children have a more pronounced stress response to surgery compared to obese children, who, at the contrary, have already at rest a high basal sympathetic activity [18].

The present results have relevant clinical consequences: In otherwise healthy schoolchildren (ASA ≤ 2) undergoing minor surgical interventions or investigations requiring anesthesia, a more prudent and economical fluid administration is warranted in order to avoid fluid accumulation (unless blood pressure and heart rate remain stable during anesthesia). One can also speculate that this category of low-risk schoolchildren do not necessitate in any case fluid administration during anesthesia.

Some limitations need to be mentioned: The preoperative and the postoperative measurements were not taken in the awake child. Nevertheless, the time delay would be potentially too long with the consequences of relevant changes during the postoperative time. Secondly, the approved study protocol did not plan to perform laboratory tests, such as measurements of electrolytes, hemoglobin, serum creatinine, albumin, or inflammatory markers, which could give us more information about the pathophysiological changes behind fluid accumulation.

Strengths of this analysis are the easy to perform, well accepted, painless, not invasive, and validated technique used to assess body composition and fluid content in children and the standardized measurement protocol, which allows precise and reproducible results.

In conclusion, this study demonstrates that fluid accumulation occurs in low-risk schoolchildren during general anesthesia. The results might suggest that children and adolescents without major health problems (ASA ≤ 2) undergoing short procedures (< 1 h) could not require any perioperative intravenous fluid therapy, unless vital parameters remain stable. Perioperative fluid prescription could be advised only in the management of critically ill children and/or long procedures, but further studies are needed. Moreover, BCM measurements yielded plausible results in children and adolescents undergoing general anesthesia and could become useful for guiding intraoperative fluid therapy in future studies.

### Supplementary Information

Below is the link to the electronic supplementary material.

Supplementary file1 (DOCX 15 KB)

## Data Availability

No datasets were generated or analysed during the current study.

## Abbreviations

ASA: American Society of Anesthesiology
BCM: Body composition monitor
BMI: Body mass index
CDC: Centre of Disease Control
ECW: Extracellular water content
ICW: Intracellular water content
TBW: Total body water
