# Supplementary material for: Fluids and body composition during anesthesia in children and adolescents: A pilot study
Source: Eur J Pediatr. 2024 Feb 26;183(5):2251–6. doi: 10.1007/s00431-024-05490-x (PMC11035464; doi:10.1007/s00431-024-05490-x)
Supplement: Supplementary file 1 — Supplementary file1 (DOCX 15 KB) [file 431_2024_5490_MOESM1_ESM.docx]

Table S1: Vital signs of 100 infants before and after anesthesia.

|  |  |  |  |  |  |
| --- | --- | --- | --- | --- | --- |
|  |  |  | **Pre** | **Post** | **p** |
|  |  |  |  |  |  |
|  |  |  |  |  |  |
| Heart Rate [beats/min] | | | 90 [75 – 102] | 86 [77.8 – 103] | 0.99 |
|  |  |  |  |  |  |
| Systolic Blood Pressure [mmHg] | | | 103.5 [93 – 108] | 94 [88 – 102] | 0.0015* |
|  |  |  |  |  |  |
| Diastolic Blood Pressure [mmHg] | | | 48 [41.8 – 63] | 44 [36 – 52] | <0.0001* |
|  |  |  |  |  |  |

Continuous variables are reported as median [25th - 75th percentile]. * p<0.05
